# School, Peer and Family Relationships and Adolescent Substance Use, Subjective Wellbeing and Mental Health Symptoms in Wales: a Cross Sectional Study

**DOI:** 10.1007/s12187-017-9524-1

**Published:** 2018-01-30

**Authors:** Graham F. Moore, Rebecca Cox, Rhiannon E. Evans, Britt Hallingberg, Jemma Hawkins, Hannah J. Littlecott, Sara J. Long, Simon Murphy

**Affiliations:** 1 Centre for the Development and Evaluation of Complex Interventions for Public Health Improvement (DECIPHer), School of Social Sciences, Cardiff University, 1-3 Museum Place, Cardiff, CF10 3BD UK; 2 Social Research and Information Division, Knowledge and Analytical Services, Health and Social Services Group, Welsh Government, Cardiff, UK

**Keywords:** Substance use, Subjective wellbeing, Mental health, Adolescence, Social relationships, Peers, Family, School

## Abstract

Positive relationships with family, friends and school staff are consistently linked with health and wellbeing during adolescence, though fewer studies explore how these micro-systems interact to influence adolescent health. This study tests the independent and interacting roles of family, peer and school relationships in predicting substance use, subjective wellbeing and mental health symptoms among 11–16 year olds in Wales. It presents cross-sectional analyses of the 2013 Health Behaviour in School-aged Children survey, completed by 9055 young people aged 11–16 years. Multilevel logistic regression analyses are used to test associations of family communication, family support, relationships with school staff, school peer connectedness, and support from friends, with tobacco use, cannabis use, alcohol use, subjective wellbeing and mental health symptoms. Positive relationships with family and school staff were consistently associated with better outcomes. Support from friends was associated with higher use of all substances, while higher school peer connectedness was associated with better subjective wellbeing and mental health. Better relationships with school staff were most strongly associated with positive subjective wellbeing, and fewer mental health symptoms where pupils reported less family support. Support from friends was associated with higher cannabis use and worse mental health among pupils with lower family support. Relationships with family and school staff may be important in protecting young people against substance use, and improving wellbeing and mental health. Interventions focused on student-staff relationships may be important for young people with less family support. Interventions based on peer support should be mindful of potential harmful effects for pupils with less support from family.

## Introduction

Adolescence is a critical life-course period for young people’s health and wellbeing (Patton et al., 2016). Many young people are exposed to psychoactive substances such as alcohol, tobacco and cannabis for the first time during this time (Donoghue et al., 2017; Hindocha et al., 2015). An estimated 1 in 5 adolescents have a diagnosable mental health problem (Green, McGinnity, Meltzer, Ford, & Goodman, 2005). Mental health problems developed during adolescence have been attributed with a tenfold greater long-term healthcare cost than those developed during adulthood (Lee et al., 2014). Mental health and subjective wellbeing have some conceptual overlap, but can be considered distinct constructs, with subjective wellbeing typically defined in terms of holistic aspects of young people’s happiness with their lives (Greenspoon & Saklofske, 1998, 2001; Keyes, 2006). While some studies indicate that subjective wellbeing is negatively correlated with mood disorders (Proctor, Linley, & Maltby, 2009) and externalizing behaviours (MacDonald, Piquero, Valois, & Zullig, 2005), recent data from the Millennium Cohort Study indicate only a weak correlation between mental health problems and subjective wellbeing in 11 year olds (Patalay & Fitzsimons, 2016). Co-morbidity of substance use with subjective wellbeing and mental health problems is however consistently demonstrated in epidemiological surveys (Lai, Cleary, Sitharthan, & Hunt, 2015), and hence, identification of common risk and protective factors is imperative in order to inform intervention approaches that simultaneously disrupt these trajectories.

According to Bronfenbrenner’s (1992) ecological systems theory, the behaviours and wellbeing of the developing person are influenced in part by biology and individual pre-dispositions, but in interaction with multiple layers of environmental influence. Within Bronfenbrenner’s model, “micro-systems” are conceptualised as those environments most proximal to the young person, which exert direct influence on health and wellbeing (e.g. schools, families and peer networks). “Meso-systems” represents the interactions between these microsystems (e.g. family-school interactions, or peer-family interactions). These are in turn nested within “exosystems” (e.g. broader education and health systems) and “macro-systems” (e.g. prevailing social and cultural norms). Finally, the “chrono-system” encompasses changes over time in young people’s interactions with, and responses to, their environments as they move through childhood and adolescence.

Influences of micro-systems such as family and friends on young people’s substance use have commonly been investigated using the Social Development Model (SDM) (Cleveland, Feinberg, Bontempo, & Greenberg, 2008). SDM hypothesises that social behaviours are learned through social interactions, which give rise to formation of attachments with lasting effect on behaviour, through supporting development of skills, norms and values (Catalano & Hawkins, 1996; Catalano et al., 2005). Attachment to others who offer opportunities for and reward prosocial behaviour protects against antisocial behaviour, while attachments to those who support and reward anti-social behaviours may increase risk behaviour (Catalano & Hawkins, 1996; Catalano et al., 2005; Schor, 1996). These relationships form much of the basis for mental health throughout adolescence and beyond (Elgar, Craig, & Trites, 2013); limited pro-social communication within families for example is linked with increased risk of mental health symptoms (Barrett, Duffy, Dadds, & Rapee, 2001; Greenberg, Domitrovich, & Bumbarger, 1999). While close relationships with family are almost universally discussed as protective for substance use, subjective wellbeing and mental health, peer relationships have been considered simultaneously as risk and protective factors. In recent analyses of data from Wales for example, young people who reported that they could count on friends reported better subjective wellbeing, though greater risk of binge drinking (Long et al., 2017).

A growing body of literature focuses on the influence of school systems on young people’s substance use and mental health, with a particular emphasis on school connectedness (Bonell et al., 2016; Markham & Aveyard, 2003). Definitions of school connectedness vary, but typically centre around student-staff cohesiveness, and relatedness to other pupils within the school setting (Markham & Aveyard, 2003; Stewart, McWhirter, Rowe, Stewart, & Patterson, 2007). Markham and Aveyard argue that structuring school social environments in a manner which minimises boundaries between teachers and pupils for example, may lead to increased engagement with norms and practices of schools, and in turn, better health and wellbeing (Markham & Aveyard, 2003; Stewart et al., 2007). The Gatehouse Project, drawing on attachment theory (Bowlby, 1998; Bowlby, 2005), focused on enhancing young people’s connectedness to school and peers, leading to improved behaviours, though not mental health outcomes (Bond et al., 2004). In a longitudinal analysis, Bond et al. (2007) found that school connectedness and connectedness to peers independently predicted mental health and substance use outcomes. More recent UK studies have replicated these associations of teacher-pupil relationships with substance use and subjective wellbeing (Long et al., 2017; Moore et al., 2017).

The impacts of these various micro-systems most likely cluster. According to attachment theory, interactions within the family environment can enable or thwart the development of capabilities to build positive relationships elsewhere (Bowlby, 2005). Young people with less supportive family environments might therefore engage less with schooling, or develop fewer good quality friendships, with cumulative effects on health and wellbeing (Bronfenbrenner, 1986). Considering independent and combined influences of these micro-systems on young people’s health and wellbeing is therefore vital in informing intervention. A recent longitudinal analysis using a global measure of social connectedness reported that high social connectedness was associated with better subjective wellbeing. While significant associations are observed across all measured domains, associations were strongest for family, followed by school, peers then community (Jose, Ryan, & Pryor, 2012).

While a growing body of studies demonstrate the importance of these micro-systems, few have moved toward understanding how interactions between them form “meso-systems” of influence on young people’s substance use and mental health. Some studies from the sociology of education have pointed to benefits of positive student-teacher interactions for educational attainment of children whose families place less emphasis on parent-child communication (Epstein, 1983). Likewise, Gorard (2010) argues that while schools cannot entirely compensate for the effects of external systems, they can provide young people with some insulation from social disadvantage, through providing opportunities for interaction with a mixed peer group and mutually respectful adult-child relationships. Few studies have tested interactions between family relationships and relationships within the school setting in predicting substance use and mental health symptoms, though it is plausible that supportive relationships with school staff may have a disproportionately beneficial influence for young people with less family support (Moore et al., 2017).

In their aforementioned longitudinal analysis, Bond et al. (2007) reported a significant interaction between school connectedness and connectedness to peers; substance use outcomes were highest among young people with low school connectedness but high connectedness to peers. Furthermore, connectedness to peers was not associated with better mental health in the absence of school connectedness. The idea that connectedness to peers may have differential impacts among subgroups of young people is supported by qualitative findings from the UK, which suggest that young people who feel disconnected from school commonly form sub-cultures whose identities are constructed around “deviant” behaviours (Fletcher & Bonell, 2013). Formalised school norms and practices may run parallel, and often in competition with, a student-led system that develops its own set of rules and regulations. Friendships may therefore serve risk or protective roles, varying according to levels of connectedness to school. Similarly, much theorisation of the interaction between family and peers in shaping young people’s behaviour has centred on the importance of risk taking in adolescent identity construction (Pound & Campbell, 2015). Where young people perceive limited closeness to family members, influence from peers may become greater than that of family members (Vitaro, Brendgen, & Tremblay, 2002). Young people who report that their parents are usually aware of their whereabouts for example are less likely to engage in substance misuse (Moore, Rothwell, & Segrott, 2010). Less positive parent-child relationships may therefore exacerbate potentially negative influences of adolescent social relationships (Martino, Ellickson, & McCaffrey, 2009).

This paper draws upon a cross-sectional school-based survey in Wales, to test the following hypotheses. First, in line with other recent studies (Jose et al., 2012), we anticipate an independent association between relationships across a range of micro-systems and young people’s health and wellbeing. More specifically, we test the hypothesis that positive family relationships, peer relationships and higher connectedness to school teachers and students will be independently associated with lowered risk of substance use and mental health symptoms, and with increased subjective wellbeing. Moving beyond a focus on individual microsystems, and toward understanding how these micro-systems combine to form meso-systems of influence (Bronfenbrenner 1992), we then test the hypotheses that i) support from teachers will play a stronger protective role for young people who report less supportive family relationships and less support from friends, ii) peer relationships will play a stronger protective role where young people also report high levels of family support.

## Methods

### Sampling, Recruitment and Data Collection

The Health Behaviour in School-aged Children (HBSC) survey in Wales 2013/14 was a cross-sectional study of young people aged 11–16 in a nationally representative sample of secondary schools in Wales (Roberts et al., 2009). Schools were also asked to complete questionnaires on the school environment and school health improvement actions (Hallingberg et al., 2016). Maintained and independent secondary schools in Wales were stratified by local authority and eligibility for free school meals, then selected using probability proportionate to size (and with an element of disproportionate stratification to allow analysis at Local Health Board level). School head teachers were invited to take part in the survey by letter and followed up with telephone calls. Overall, 181 schools were invited to take part in order to reach the target sample size of 82 schools. Participating schools received £150 to cover any costs incurred due to participating. Ethical approval was obtained from the Cardiff University School of Social Sciences Research Ethics Committee. Within each participating school (*n* = 82), one mixed ability class (approximately 25 students) from each school year 7–11 was randomly selected to participate. Data were collected between November 2013 and February 2014. Trained fieldworkers attended each data collection to ensure sufficient support and assistance where required. Teachers were present during data collection but remained at the front of the classroom so they could not see students’ responses.

### Measures

#### Socioeconomic Status

Welsh Government data on the percentage of students eligible for free school meals (FSM), due to their parents receiving income support, is included as a measure of school-level SES. The Family Affluence Scale (Currie et al., 2008) measured family level SES. This was a sum score of survey items assessing car and computer ownership, frequency of holidays and bedroom occupancy. For the 2013/14 HBSC survey, due to concerns that some items within FAS were losing their saliency as markers of affluence (i.e. computer ownership) an additional two items were added relating to dishwasher ownership and the number of bathrooms in the home. Aggregated at the school level, the full scale with these additional items demonstrated a stronger association with FSM (*r* = 0.80) than the original four item FAS scale (*r* = 0.67).

#### Family Relationships

Young people answered questions about relationships with family, including perceptions of the extent to which important things were talked about, that their family tries to help them and provision of emotional support. A factor analysis indicated that items loaded onto two separate factors, and were thus used as two unique variables: one relating to family communication, and one relating to family (social and emotional) support, with Cronbach’s alpha statistics of 0.86 and 0.95 respectively.

#### Support from Friends

Young people were asked to indicate their agreement or disagreement with 4 items on a 7-point Likert scale, relating to the extent to which they felt that friends tried to help them, could be counted on when things go wrong, could share in joys and sorrows, and that they could talk easily to friends. All items loaded onto a single factor, with good internal consistency (alpha = 0.92).

#### School Connectedness

Three questions on a 5-point Likert scale asked students to rate the extent to which they felt accepted by their teachers, that their teachers cared about them as a person and that they trusted their teachers. The items demonstrated good internal consistency and were summed to form a single measure of “teacher support” (alpha = 0.80). Three further questions on a 5-point Likert scale asked students whether they felt students in their class enjoyed being together, were kind and helpful and accepted them as they were. The items demonstrated acceptable internal consistency and were summed to form a single measure of “peer connectedness (within school)” (alpha = 0.69).

#### Substance Use

A single item asked young people how often they had drunk alcohol in the past 30 days. Any score other than “never” or “1–2 times” was considered regular drinking. Smoking was assessed by asking young people how often they currently smoked, with response options of “I do not smoke”, “less than weekly”, “weekly” or “daily”. Analyses were repeated with two derived binary variables, one defining only those who smoked weekly or more as smokers, and one including all responses other than never. As results were consistent, only analyses for weekly smoking are presented. A single item asked young people how often they had used cannabis in the past 30 days. Young people providing any response other than “never” were considered cannabis users. Young people were also asked whether they had ever taken any of a list of substances at least once as a measure of ever drug use.

#### Subjective Wellbeing

To measure subjective wellbeing, young people were asked to indicate on a scale of 0 to 10, how satisfied they were with their life. Due to a high degree of skewness, this measure was divided into approximately equal tertiles for analysis (0–6; 7–8; 9–10).

#### Mental Health Symptoms

Mental health symptoms were measured via a list of items relating primarily to internalising problems used in previous international HBSC analyses (Elgar et al., 2013), asking young people to indicate how often they felt low, irritable, anxious or had trouble sleeping. All items loaded onto a single factor with good internal consistency (alpha = 0.79). Due to a high degree of skewness, this item was divided at the median to form a binary outcome variable.

### Statistical Analysis

First, Spearman’s Rank Correlation coefficients assessed associations between family, peer and school measures, and between dependent variables (substance use, mental health symptoms and subjective wellbeing). To facilitate examination of the correlation between substance use and mental health symptoms and wellbeing, a composite measure of substance use was created by summing the binary items to form a scale of 0–3 (i.e. user of none of the above substances vs user of all 3). Subsequently, multivariate mixed effects logistic regression models (adopted due to the clustered nature of the data sample and the degree of intra-cluster correlation in key variables) were used to test the independent associations of family, peer and school measures with all dependent variables. Third, for all dependent variables, interaction terms were fitted testing the interactions between: support from friends*support from family, teacher support*support from friends and support from family*teacher support. Post-hoc sub-group analyses aided interpretation of any significant interactions, with multi-level models rerun for young people with higher or lower than average family support and higher or lower than average support from friends. Models for all substance use variables and mental health symptoms were binary logistic models, while subjective wellbeing was divided into tertiles and subjected to ordinal logistic regression analyses. All models are adjusted for year group, gender family affluence and FSM entitlement. While use of survey weights in multi-level analysis requires weights at each level of analysis (i.e. school and pupil), only a single combined weight was available in the HBSC dataset. However, weighting did not significantly alter population estimates for any of the variables of interest, while single-level regression analyses with or without weighting produced consistent estimates. Hence, unweighted multi-level models are presented.

## Results

### Sample Descriptions

A total of 39 young people were withdrawn from the study by parents, 33 refused to participate, and 772 were absent on the day of data collection, with data obtained from 9055 students (91.5% of eligible pupils). Demographic variables are presented below in Table 1. The vast majority of young people were categorised as non-substance users (*N* = 7394, 89.7%), with 7.5% (*N* = 615) reporting use of one substance, 1.9% (*N* = 164) reporting two and less than 1% reporting use of all three (*N* = 71, 0.9%). Mental health symptoms and subjective wellbeing were moderately inversely correlated with one another (*r* = −0.42, *p* < 0.01), while a higher composite substance use score was more weakly associated with increased mental health symptoms (*r* = 0.20, p < 0.01) and reduced subjective wellbeing (*r* = −0.13, p < 0.01).

**Table 1:** Sample descriptions

	N	Mean (SD)/N(%)
Mean (SD) age	9010	13.7 (1.4)
N (%) female	9022	4457 (49.4)
Mean (SD) FAS (summed 6 item scale)	8779	15.1 (2.3)
Mean (SD) FSM	9055	14.9 (8.4)
N (%) smokers (occasional and weekly or more)	9029	465 (5.2)
N (%) smokers (weekly or more)	9029	299 (3.3)
N (%) frequent alcohol drinkers	8577	691 (8.1)
N(%) cannabis use in past month	8662	249 (2.9)
N(%) ever use of any drug	8325	679 (8.2)
Mean (SD) subjective wellbeing	8721	7.3 (1.9)
Mead (SD) mental health symptoms	8797	4.6 (4.1)

### Associations between Family, Peer and School Measures

All peer, family and school variables were significantly and positively associated with one another (see Table 2). Teacher support was strongly associated with connectedness to peers in school, though only moderately correlated with support from friends. Higher levels of family support were associated with support from friends and better school connectedness.

**Table 2:** Spearman’s rank correlation coefficients for associations between family, friendship and school relationship variables (*N* = 7594). All associations are significant (*p* < 0.05)

	Family communication	Family support	Teacher support	Support from friends
Family support	0.46			
Teacher support	0.35	0.20		
Support from friends	0.19	0.56	0.11	
Peer connectedness (school)	0.34	0.18	0.45	0.19

### Associations of Family, Peer and School Measures with Substance Use, Wellbeing and Mental Health

Results of logistic regression analyses are presented in Table 3. All independent variables relating to family and teacher relationships were significantly, and independently, associated with substance use, subjective wellbeing and mental health. Peer connectedness in the school environment was associated with better wellbeing and mental health, though not with substance use. Conversely, support from friends was associated with increased risk of smoking, cannabis use, drinking and drug use.

**Table 3:** Odds ratios and 95% confidence intervals for associations of relationship variables with substance use, subjective wellbeing and mental health symptoms

	Smoking (*N* = 7357)	Cannabis use (*N* = 7081)	Drug use (*N* = 6834)	Alcohol use (*N* = 7056)	Subjective Wellbeing (*N* = 7232)	Mental health symptoms (*N* = 7253)
*Main effects*						
Teacher support	**0.61**	**0.55**	**0.63**	**0.72**	**1.31**	**0.90**
	**(0.52, 0.71)**	**(0.46, 0.64)**	**(0.57, 0.70)**	**(0.65, 0.80)**	**(1.24, 1.38)**	**(0.84, 0.95)**
Family communication	**0.82**	**0.75**	**0.78**	0.92	**1.95**	**0.61**
	**(0.71, 0.94)**	**(0.61, 0.92)**	**(0.70, 0.86)**	(0.83, 1.02)	**(1.84, 2.07)**	**(0.58, 0.65)**
Family support	**0.74**	**0.79**	**0.78**	**0.74**	**1.15**	**0.87**
	**(0.61, 0.89)**	**(0.68, 0.92)**	**(0.69, 0.89)**	**(0.65, 0.84)**	**(1.08, 1.23)**	**(0.81, 0.94)**
Support from friends	**1.41**	**1.34**	**1.24**	**1.34**	1.06	0.96
	**(1.18, 1.70)**	**(1.11, 1.63)**	**(1.10, 1.41)**	**(1.19, 1.52)**	(0.99, 1.14)	(0.90, 1.04)
Peer connectedness (in school)	1.11	1.06	1.06	1.00	**1.43**	**0.69**
	(0.94, 1.31)	(0.90, 1.27)	(0.95, 1.19)	(0.89, 1.12)	**(1.34, 1.52)**	**(0.65, 0.74)**
*Interactions*						
Family support * Teacher support	0.93	0.90	1.02,	1.03	**0.93**	**1.06**
	(0.81, 1.06)	(0.78, 1.04)	(0.93, 1.12)	(0.95, 1.13)	**(0.89, 0.98)**	**(1.01, 1.12)**
Teacher support * Support from friends	0.99	1.14	1.05	**1.10**	**0.92**	1.03
	(0.85, 1.14)	(0.97, 1.33)	(0.95, 1.15)	**(1.00, 1.21)**	**(0.88, 0.97)**	(0.98, 1.09)
Family support * Support from friends	0.88	**0.79**	0.90	0.95	**1.50**	**0.70**
	(0.74, 1.05)	**(0.66, 0.95)**	(0.81, 1.02)	(0.85, 1.06)	**(1.41, 1.59)**	**(0.65, 0.74)**

Bold print indicates p < 0.05

There were significant interactions between family support and teacher support for subjective wellbeing (OR = 0.93) and mental health symptoms (OR = 1.06), though not for substance use consistent with a hypothesis that associations of teacher support with wellbeing and mental health are stronger for pupils with less family support (see post-hoc analyses below). Likewise, the interaction between teacher support and support from friends was significant for subjective wellbeing (OR = 0.92), but not for mental health symptoms. An interaction between support from family and support from friends ran in the opposing direction, consistent with a hypothesis that associations of support from friends with wellbeing (OR = 1.50) and mental health (OR = 0.70) are strongest where support from family is also highest. For substance use, the only significant interactions were between family support and support from friends in predicting cannabis, and for alcohol, between teacher support and support from family.

### Post Hoc Tests of Subgroup Effects

In separate models for young people with ‘high’ and ‘low’ family support, teacher support was associated with significantly improved subjective wellbeing in both models, though with a higher odds ratio among young people with low family support (OR = 1.40; CI = 1.28 to 1.54; *N* = 2599) than those with high family support (OR = 1.22 CI = 1.13 to 1.31; *N* = 4633). Hence, findings are consistent with an interpretation that teacher support is associated with better wellbeing for both groups, though more strongly for pupils with less family support. For mental health symptoms, teacher support was associated with significantly lower odds of mental health symptoms for young people with low family support (OR = 0.89; CI = 0.80 to 0.99; *N* = 2625) though not for young people with high family support (OR = 0.95; CI = 0.87 to 1.02; *N* = 4628). Hence, data are consistent with an interpretation that teacher support is associated with a lowered risk of mental health symptoms only for pupils with less family support.

For support from friends, there was evidence of a positive association with subjective wellbeing for young people with high levels of family support only (OR = 1.39; CI = 1.26 to 1.54; N = 4633), with no significant association (and indeed a negative coefficient) for young people with lower family support (OR = 0.95; CI = 0.86 to 1.05). Furthermore, associations of support from friends with mental health symptoms operated in opposing directions according to level of family support. Higher support from friends was associated with an increased risk of mental health symptoms among young people with low family support (OR = 1.17; CI = 1.04 to 1.32; *n* = 2625), though with a reduced risk of mental health symptoms among young people with high family support (OR = 0.73; CI = 0.66 to 0.81; *n* = 4628). For cannabis use, support from friends was associated with increased risk of cannabis among young people with low family support (OR = 1.57; CI = 1.20 to 2.06; *n* = 2560) though not among young people with high family support (OR = 0.92; CI = 0.64 to 1.31; *n* = 4539). Hence, data are consistent with an interpretation that support from friends may be associated with more positive outcomes for young people with high levels of family support, though with neutral, or negative, outcomes for pupils with less family support.

In models for young people with relatively high and low support from friends, teacher support was associated with higher subjective wellbeing for both groups, though with a stronger association for young people with low (OR = 1.36; CI = 1.25 to 1.49; *N* = 2988), rather than high support from friends (OR = 1.25; CI = 1.15 to 1.34; *N* = 4244). Similarly, teacher support was associated with reduced risk of alcohol use for both groups, though with a stronger association for young people with low (OR = 0.65; CI = 0.55 to 0.76; *N* = 2917), rather than high support from friends (OR = 0.81; CI = 0.70 to 0.93; *N* = 4139). Hence, findings are consistent with an interpretation that teacher support is associated with reduced risk of alcohol consumption to a greater extent among young people with less support from friends.

## Discussion

Consistent with previous literature (Bond et al., 2007; Jose et al., 2012), this paper demonstrates significant associations between young people’s interactions with a range of microsystems (Bronfenbrenner, 1986, 1992) and their health and wellbeing outcomes. Positive relationships with teachers and support from family were consistently associated with significantly better mental health and subjective wellbeing, and with lower risk of substance use. The role of peers was however somewhat more ambiguous, including both positive and negative associations with health and wellbeing outcomes. In contrast to some previous studies (Bond et al., 2007), though consistent with recent studies from Wales (Long et al., 2017), supportive relationships with friends were associated with increased risk of smoking, drinking and drug use, though not subjective wellbeing and mental health. By contrast, young people who reported better connectedness to peers in the school environment reported better wellbeing and mental health (though did not differ in terms of substance use risk).

Hence, these findings are supportive of a hypothesis that substance use and mental health symptoms share common risk factors in terms of the roles of family and school teachers, though with greater divergence between outcomes in the nature of peer friendships. Perceptions of support from family, friends and school staff tended to cluster, with significant positive correlations between them. As the home environment plays a significant role in providing young people with the capabilities to develop healthy relationships outside of the family environment, effects of poor family relationships may perhaps be compounded by knock on effects for relationship formation beyond the home (Bowlby, 1998; Bowlby, 2005). Consistent with recent UK studies however, these various micro-systems were independently associated with wellbeing outcomes in multivariate models (Jose et al., 2012).

The paper extends previous studies focused on environmental influences on adolescent wellbeing through focusing on meso-systemic influences on young people’s substance use and mental health; consistent with Bronfenbrenner’s (1992) ecological systems theory, there were a range of interactions between family, school and peer micro-systems. Peer relationships and school connectedness interacted with family relationships, in opposing directions, in relation to subjective wellbeing and mental health symptoms. Relationships with school staff were more positively associated with mental health and subjective wellbeing for young people with less family support, consistent with a hypothesis that support from teachers may be most beneficial for young people with less supportive family environments. Hence, Markham and Aveyard’s conceptualisations of health promoting schools as those who structure their social environments in a manner which minimises boundaries between teachers and pupils may be particularly important for young people with less family support (Markham & Aveyard, 2003; Stewart et al., 2007). A disproportionately protective association for educational attainment outcomes of teacher relationships for young people with low levels of maternal attachment has been reported in North American studies (O’Connor & McCartney, 2007), though has not previously been tested in relation to health and wellbeing to our knowledge.

In addition, the study indicated that the nature of association of support from friends with some outcomes ran in opposing directions, according to the degree of family support. For young people who reported less family support, a high degree of support from friends was associated with increased mental health symptoms, as well as a higher risk of cannabis use. Where young people perceive limited closeness to family members, influence from peers may become greater than that of family members (Moore et al., 2010; Vitaro et al., 2002). Lower levels of family support may therefore exacerbate the more negative influences of adolescent social relationships on young people’s health and wellbeing (Martino et al., 2009). Conversely, support from friends may have a positive influence on young people’s wellbeing where accompanied by support from family. Data from the Gatehouse Project in Australia (Bond et al., 2007) found that connectedness to peers was associated with improved mental health only for pupils with higher levels of connectedness to school staff; an interaction not replicated in this study. In the present study support from teachers was associated with improved subjective wellbeing for young people with high or low support from friends, though with associations strongest among pupils with lower levels of support from friends.

The study benefits from a large sample of young people in Wales. However, it has a number of important limitations. First, it is based on cross sectional data and hence, cannot demonstrate cause and effect; young people with better mental health outcomes and fewer behaviours which transgress school norms, such as drug use, may find it easier to build relationships with school staff. Second, the use of a secondary dataset for analysis not designed for this purpose means that the measures of connectedness to school, family and peers are not directly comparable. Differences in strength of associations across domains may in part reflect differences in measures, rather than the relative importance of those micro-systems. Survey weights were not employed in the analysis, although sensitivity analyses indicated that estimates and associations were robust whether or not survey weights were applied. Finally, the measures of mental health and wellbeing included within the survey are simplistic given the complex and multi-dimensional nature of these constructs. There is not clear consensus on how to best measure subjective wellbeing in young people; in this study, an evaluative measure of global wellbeing was used (life satisfaction), as opposed to an experience (momentary affect) or eudemonia (purpose) measure (Dolan, Layard, & Metcalfe, 2011; Dolan & Metcalfe, 2012). The reliability of evaluative measures is more clearly established than other constructs of wellbeing, although is higher for composite measures than for the kind of single item measure used in this study (Kapteyn, Lee, Tassot, Vonkova, & Zamarro, 2015). The generalisability of observed associates across alternative dimensions or measurement approaches for adolescent mental health or subjective wellbeing requires further investigation.

Nevertheless, the study has important potential implications for interventions to reduce young people’s uptake of substance use and improve mental health and wellbeing outcomes. Intervention based on supporting the formation of healthy relationships within both the home and school environments may play important roles in reducing substance use, improving subjective wellbeing and mental health. Interventions centred around promoting school connectedness should pay close attention to how their effects are moderated by relationships within the family environment. Interventions centred on the formation of supportive peer relationships perhaps need to monitor the potential for harmful effects, and develop strategies to overcome them, where friendships become the primary source of support for young people in the absence of support from family or adults within the school environment. Further longitudinal research is needed to understand the causal nature of the associations investigated in this study.
